# From Classical Bacterins to Recombinant Vaccines: Critical Aspects of the Immune Response in Ruminants

**DOI:** 10.3390/microorganisms14040790

**Published:** 2026-03-31

**Authors:** Juliana Loria, Cynthia Baldwin, Walter Lilenbaum

**Affiliations:** 1 Laboratory of Veterinary Bacteriology, Biomedical Institute, Federal Fluminense University, 101 Prof. Hernani Melo Street, Niteroi 24210-130, RJ, Brazil; julianaloria@id.uff.br; 2 Department of Veterinary and Animal Sciences, University of Massachusetts, Amherst, MA 01003, USA; cbaldwin@vasci.umass.edu

**Keywords:** chronic disease, leptospirosis, sterilizing immunity, Th1 immune response, γ*δ* T cells

## Abstract

Leptospirosis is a neglected zoonosis causing significant economic losses in livestock, primarily through Bovine Genital Leptospirosis (BGL). While current vaccines prevent clinical disease, they typically fail to provide sterilizing immunity against adapted strains. This allows *Leptospira* to persist in the genitourinary tract, maintaining environmental shedding and zoonotic risk. Achieving sterilizing immunity remains a challenge, and this gap may be closely related to the immune response pattern of ruminants, where effective protection against chronic colonization requires, besides the humoral response, a robust cellular immune response (Th1/IgG2). Recent studies indicate that adjuvants based on oil emulsions or biodegradable polymers are better at inducing Th1/IgG2 responses and the proliferation of CD4^+^ T cells, as well as WC1^+^ γ*δ* T cells, which may be essential for eliminating *Leptospira* from renal and probably also genital tissues. Thus, overcoming chronic colonization through inducing the Th1-type immune response may be the main challenge for vaccination to fulfill its role in sustaining herd immunity and mitigation of zoonotic risk, in line with the One Health approach. In this context, we aimed to critically examine immune mechanisms in ruminants, advances in vaccine platforms and adjuvant strategies against bovine leptospirosis and outline the challenges that must be overcome to achieve sterilizing immunity.

## 1. Introduction

Leptospirosis is a neglected zoonotic disease caused by pathogenic bacteria in the genus *Leptospira*, affecting humans as well as various domestic and wildlife species [1]. Although globally distributed, the disease disproportionately impacts tropical and subtropical regions. In these areas, environmental drivers such as heavy rainfall, flooding, and inadequate sanitation facilitate transmission and triggering of seasonal outbreaks in both rural and urban environments [2,3]. Recent studies highlight the impact of climate change and extreme weather events in exacerbating the incidence of leptospirosis, expanding its geographic range and reinforcing its relevance as a re-emerging global health threat [4,5,6]. In this context, integrated One Health frameworks are increasingly recognized as vital for linking animal infection, environmental contamination, and human health risks, particularly in the development of vaccine strategies aimed at mitigating zoonotic spillover [2,7,8,9].

Ruminants, especially cattle, play a central role as maintenance hosts, perpetuating the infection through persistent renal and genital colonization [10,11]. Thus, these features make leptospirosis one of the most important causes, not only of reproductive and economic losses in livestock production systems [11,12], but also of public health risks. The establishment of this chronic carrier state is a direct consequence of the pathogen’s ability to colonize immunologically privileged sites, such as the proximal renal tubules and the genital tract, where it remains protected from systemic defense mechanisms [11,12,13].

The global burden of ruminant leptospirosis is substantial. Recent meta-analyses report a pooled anti-*Leptospira* seroprevalence of 40% in cattle and approximately 19% in goats, with regional rates reaching 44% in Europe and 34% in North America [14,15]. Furthermore, epidemiological studies emphasize the role of bull urine and semen as potential vectors for *Leptospira*, reinforcing the need for greater control strategies [16,17]. These infections result in major economic disruptions, primarily through reproductive failures such as abortion, stillbirth, and infertility [11,14], and effectively addressing this burden requires vaccines capable of inducing sterilizing immunity to break the transmission cycle.

Despite decades of research, developing a vaccine that offers long-lasting, cross-protective immunity among the various pathogenic serovars and sterilizing immunity remains a challenge for the global control of this disease [13,18,19]. In this context, sterilizing immunity is defined as the complete prevention of infection, encompassing the elimination of renal and genital colonization and the total cessation of bacterial shedding in urine and reproductive fluids [19].

Current bacterins, consisting of inactivated whole leptospires, provide only serovar-specific protection. While these formulations induce high titers of agglutinating antibodies (predominantly IgG1) that prevent acute systemic disease, they often fail to prevent genitourinary colonization [13,20]. Evidence suggests this shortcoming may be linked to their inability to activate the specific cellular immune axes required for tissue clearance in maintenance hosts [20,21]. In particular, the frequent use of aluminum-based adjuvants tends to favor a Th2-biased humoral response, which may not be sufficient to stimulate interferon-gamma (IFN-γ) production by the expansion of WC1^+^ γδ T cells and CD4^+^ Th1 cells that produce this cytokine. However, while a Th1-oriented cellular response, involving WC1^+^ γδ T cells and IgG2 production, appears to be an important component associated with tissue clearance, this mechanism should be considered a correlate of protection rather than a definitively established requirement in all contexts. Without these aspects of the immune response, vaccinated animals may remain susceptible to chronic shedding, thereby contributing to environmental dissemination [21,22].

Emerging strategies emphasize the selection of conserved antigens and the employment of innovative platforms, including DNA, mRNA, and recombinant proteins, which may be able to overcome these limitations. These technologies, supported by immuno-informatics and reverse vaccinology (RV), allow for the systematic screening of the bacterial genome to identify candidates with a high potential. By fostering coordinated humoral and cellular responses, these recombinant approaches represent a promising pathway toward achieving sterilizing immunity and controlling leptospirosis in chronically infected cattle [23,24] when combined with the new candidates.

## 2. Leptospirosis in Ruminants

### 2.1. Pathogenesis

The clinical presentation of leptospirosis is primarily dictated by the relationship between the host and the infecting serovar. It is essential to differentiate between acute infection, typically caused by incidental serovars, and the chronic carrier state associated with host-adapted serovars. Acute leptospirosis is characterized by leptospiremia and systemic manifestations such as fever, hemolytic anemia, and hepatic or renal dysfunction, occurring more frequently in young animals or incidental hosts. In the Americas, incidental serovars of *Leptospira* included in multivalent bacterins vary according to regional epidemiology, with Pomona, Grippotyphosa, Icterohaemorrhagiae, and Canicola commonly used in North America and thus these are considered to be the main cause of acute leptospirosis [21,25].

Conversely, the chronic carrier state is the hallmark of infection by adapted serovars, most notably those belonging to the Sejroe serogroup. In these cases, the infection is often subclinical, yet it leads to significant reproductive failures, including infertility, embryonic death, stillbirths, and abortions [11,12]. While vaccines are generally effective in controlling the systemic signs of acute disease, they often fail to prevent the establishment of chronic colonization by adapted strains, which remains a critical challenge for herd management [13,19].

### 2.2. Mechanisms of Renal and Genital Colonization

The persistence of *Leptospira* spp. in ruminant hosts is driven by complex interactions that allow the bacteria to colonize immune-privileged niches. After the transient systemic phase, leptospires migrate to the proximal renal tubules and the uterine mucosa. In these environments, the bacteria escape systemic immune surveillance [13,26].

The mechanisms of colonization involve a specialized repertoire of virulence factors. Among these, Virulence Modifying (VM) proteins (encoded by the *PF07598* gene family) have been identified as key players in modulating the initial host inflammatory response, facilitating bacterial evasion and local persistence [27,28,29]. By establishing themselves in the renal interstitial tissue and reproductive tract, leptospires can persist for months or even years. This leads to intermittent shedding in the urine and vagina, which ensures the maintenance of the bacteria within the herd and environmental contamination [11,12,30].

### 2.3. Serovar Diversity and Host Adaptation

The epidemiology of leptospirosis in ruminants is increasingly complex due to high serovar diversity and shifting adaptation patterns. While *L. interrogans* serovar Hardjo (genotype Hardjoprajitno) and *L. borgpetersenii* serovar Hardjo (genotype Hardjobovis) remain the primary agents of Bovine Genital Leptospirosis (BGL) globally, other strains are gaining prominence. Recent molecular studies have highlighted the role of *L. santarosai* serovar Guaricura as a significantly adapted agent in South American herds [14,31,32].

This diversity is further complicated by the interaction between livestock and wildlife, which acts as a reservoir for incidental strains. Genomic analyses indicate that while core proteins like LipL32 and Loa22 are highly conserved, vaccine-relevant antigens such as LigA and OmpL1 exhibit significant sequence variation among different lineages. This antigenic variability may explain the limited cross-protection provided by standard bacterins and underscores the need for localized, genome-informed vaccine development [33].

### 2.4. Diagnostic Challenges for Ruminant Leptospirosis

Diagnosing leptospirosis in ruminants, particularly in chronic carriers, also presents substantial technical hurdles. The Microscopic Agglutination Test (MAT), the gold standard for serology, often yields low or even negative results of antibody titers in animals infected with host-adapted strains. Furthermore, MAT results are frequently confounded by vaccine-induced antibodies, making it difficult to distinguish between infected and immunized animals [34,35].

Consequently, there is an urgent need for the integration of molecular methods alongside MAT, such as PCR in urine and cervical–vaginal mucus, to accurately identify maintenance hosts in the herd. However, although molecular tools are effective, their high cost and the requirement for specialized laboratories or equipment often preclude their routine use in field conditions [35]. In this context, there is an urgent need for validated point-of-care (POC) diagnostic tools that can reliably identify asymptomatic carriers at the farm level, facilitating more targeted control strategies [36].

### 2.5. Limitations of Current Control Strategies

Vaccination remains the cornerstone of leptospirosis control in livestock. Despite the widespread use of vaccines, current formulations, primarily based on inactivated whole-cell bacterins, offer limited protection [13,19,37]. Furthermore, the protection provided is typically serogroup-specific and short-lived, requiring frequent boosters [18,20].

The virulence of the strains also used in vaccine production significantly influences efficacy. Bacterins derived from virulent strains tend to elicit superior immune responses compared to those using highly passaged, attenuated strains [38]. To overcome these shortcomings, current research is directed toward identifying novel antigens and adjuvants capable of stimulating both humoral and robust cellular (Th1-type) immune responses, which are essential for clearing persistent colonization [26,39].

In this sense, understanding innate and adaptive immunity is fundamental for the development of new vaccines that overcome the shortcomings of current technologies [26,39]. However, understanding the antigen diversity may require advanced bioinformatics insights and clinical validation of novel antigens, a process mirroring the genome-wide characterization approaches used for other complex allergens and pathogens [40].

## 3. Immune Responses to *Leptospira* Infection in Ruminants

The immune response in ruminants is a complex orchestration of innate and adaptive mechanisms, which could vary significantly depending on whether the host is facing an incidental or a host-adapted serovar of *Leptospira*. Understanding these nuances is essential for differentiating between protection against acute clinical disease and the prevention of chronic persistence in the genitourinary tract [12,20,26].

*Leptospira* infection begins with invasion of the bloodstream (leptospiremia) from mucous membranes or damaged skin. The innate immune system serves as the first line of defense, utilizing pattern recognition receptors (PRRs) to identify leptospiral ligands. A distinctive feature of *Leptospira* is that its lipopolysaccharide (LPS) is primarily recognized by TLR2 rather than TLR4 in many species, including humans and potentially ruminants. This structural variation likely facilitates immune evasion by avoiding the classical TLR4-mediated inflammatory cascade [39,40,41]. Beyond LPS, surface lipoproteins such as LipL32 and other transmembrane proteins activate the TLR2/TLR1 heterodimer, triggering the secretion of pro-inflammatory chemokines like IL-8 and MCP-1, which recruit neutrophils and monocytes to the site of infection [42,43].

Recent findings highlight the role of intracellular receptors, particularly NOD2, which has been linked to the development of innate immune memory, or so-called “trained immunity.” This process may shape subsequent adaptive responses and influence the host’s ability to limit the initial bacterial load. However, while initial studies in murine models suggest a role for trained immunity in leptospirosis, it is crucial to note that the specific epigenetic mechanisms in bovine γδ T cells being trained remain to be fully characterized under field conditions, and specifically we do not know whether this occurs in response to leptospirosis before this can be considered to have a definitive role in a vaccine roadmap [44,45,46,47].

Pathogenic strains have evolved sophisticated evasion strategies, such as the production of proteins (e.g., LIC_10499 and LIC_12339) that interact with host regulatory factors like C4BP and Factor H to inhibit the complement cascade [43]. The modulation of the NLRP3 inflammasome also appears to be a key factor; while its activation is necessary for initiating inflammation, adapted strains may manipulate this pathway to favor a low-inflammatory environment conducive to chronicity [42,44].

The adaptive response is conventionally divided into humoral and cellular axes. The humoral response, characterized by a Th2-type profile and the production of agglutinating antibodies (primarily IgM and IgG1), is the principal mechanism for controlling acute leptospiremia. In incidental infections, these antibodies effectively clear the pathogen from the bloodstream, thereby preventing systemic dissemination and acute clinical signs [13,20]. Current commercial bacterins are potent inducers of this humoral axis, particularly when formulated with aluminum hydroxide adjuvants, which promote dendritic cell recruitment and prolonged CD4^+^ T cell interaction [48,49]. While these vaccines are successful in reducing acute clinical disease, they often fail to achieve sterilizing immunity [21,26].

The eradication of *Leptospira* from immunologically privileged sites, such as the proximal renal tubules and the reproductive tract, appears to require a robust Th1-type cellular immune response. In cattle, this axis is defined by the production of interferon-gamma (IFN-γ) and the IgG2 subclass of antibodies, which enhances opsonophagocytic and the activation of macrophages [50,51].

Evidence suggests that the requirement for a strong Th1/IgG2 axis is most critical in the context of host-adapted serovars (e.g., Hardjo). While agglutinating antibodies may contribute to limiting the initial systemic phase, the cellular response is hypothesized to be the primary driver for preventing persistent colonization [20]. This distinction is vital for vaccine design: while preventing acute disease relies on early antibody titers, preventing chronic BGL likely requires antigens and adjuvants capable of stimulating long-term cellular memory, such as VM proteins or biodegradable polymers [27,52].

A unique and defining feature of the ruminant immune system is the high frequency of γδ T cells, which can constitute up to 60% of circulating lymphocytes in young animals. A specific subpopulation expressing the Workshop Cluster 1 (WC1) coreceptor is particularly reactive to leptospiral protein antigens [53,54]. The WC1 molecules on subpopulations of γδ T cells act as both PRRs and co-receptors for the T-cell receptor (TCR), proliferating and producing significant amounts of IFN-γ upon exposure to *Leptospira* [22,55,56,57].

Research indicates that the γδ T cell response may precede and direct the subsequent CD4^+^ Th1 response, serving as a bridge between innate and adaptive immunity [58]. Moreover, these cells exhibit characteristics of “trained immunity,” showing enhanced recall responses that can persist for months after vaccination [59,60]. However, the response is balanced; some γδ T cell populations may perform regulatory functions through IL-10 secretion, potentially influencing the transition from acute inflammation to bacterial persistence [61]. The expansion of these cells is now considered a key correlate of cellular protection in ruminant vaccine trials [20,22].

In summary, for adequate control of BGL, it is essential to distinguish the mechanisms necessary for different protective outcomes. While the prevention of acute leptospiremia is primarily mediated by the innate inflammatory response and agglutinating antibodies that neutralize the pathogen during its systemic phase, the prevention of renal colonization appears to require a transition to a Th1/IgG2 cellular profile with activation of γδ T cells to eliminate bacteria from tissues [20,51,62]. Table 1 provides a comparative overview of these immune response profiles following vaccination. Effective protection against *Leptospira* is likely a continuum requiring both humoral and cellular components, with their relative weight shifting according to the disease phase and the infecting serovar.

While this Th1/IgG2 framework is highly plausible and supported by the relevant literature, it should be accepted with some caution. For example, the cellular response is increasingly supported as a key component of the bovine immune response, but its role as the “true” or “only” mechanism of protection against chronic colonization is not yet definitively established in all contexts [20,63]. What is known is that ruminants utilize a combination of humoral and cellular mechanisms to combat *Leptospira*, with CD4^+^ and γδ T cells and Th1-type cytokines being prominent features of their immune repertoire. What remains uncertain is the exact threshold of cellular activation required to achieve sterilizing immunity, especially in the genital tract, and the degree to which these mechanisms can be generalized across different serovars [64]. This means that future vaccine design may need to aim for a balanced response by both axes while prioritizing the induction of long-term cellular memory.

## 4. Classical Bacterins to Recombinant Vaccines and the Challenge of Promoting Sterilizing Immunity Against Leptospirosis

The evolution of leptospiral vaccines reflects a progressive transition from empirically derived whole-cell formulations to rationally designed molecular strategies, driven by the need to overcome the intrinsic limitations of conventional bacterins [13,18,19]. Despite being developed decades ago, bacterins remain the cornerstone of leptospirosis control in livestock due to their affordability and effectiveness in preventing acute disease, reproductive losses, and mortality [21]. However, their protective scope is inherently restricted. Immunity is predominantly directed against lipopolysaccharide (LPS), resulting in a serogroup-specific and short-lived response that frequently requires periodic revaccination [18,20]. Although certain formulations, particularly those adjuvanted with aluminum hydroxide, have demonstrated partial efficacy in reducing renal colonization and bacterial shedding under specific conditions [13,30,65], these effects may be insufficient to prevent the establishment of chronic carrier states [18,20,21]. Moreover, bacterin-induced antibodies interfere with serological diagnostics such as the Microscopic Agglutination Test (MAT), complicating the differentiation between infected and vaccinated animals [34,35].

These limitations are particularly critical in the context of the increasingly complex epidemiology of leptospirosis in ruminants. Molecular studies have highlighted the emergence and adaptation of strains such as *Leptospira santarosai* serovar Guaricura in South American herds [16,32], reinforcing the inadequacy of serovar-restricted vaccines in dynamic epidemiological settings. Although core leptospiral proteins are relatively conserved, key vaccine targets, including LigA, exhibit sequence variability that may compromise cross-protection [33]. Consequently, accurate identification of maintenance hosts and transmission dynamics increasingly depends on the integration of molecular diagnostics, such as PCR, alongside traditional serology [12,35].

At the host–pathogen interface, the persistence of *Leptospira* in renal and reproductive tissues, particularly in the context of bovine genital leptospirosis (BGL), is driven by a complex interplay between immune evasion mechanisms and host immune responses [11,12]. Mathematical modeling studies reinforce the concept that asymptomatic carriers are central to transmission dynamics, especially under conditions of imperfect vaccination [21]. In this scenario, vaccines that fail to induce sterilizing immunity may inadvertently contribute to the silent circulation of the pathogen, masking infection within herds while sustaining zoonotic risk and production losses [21].

Mechanistically, leptospiral persistence is closely associated with modulation of innate immunity. The atypical structure of leptospiral LPS alters signaling through pattern recognition receptors, including Toll-like receptors, affecting inflammasome activation, and promoting immune evasion strategies such as macrophage apoptosis [40]. In parallel, pathways such as NOD2-mediated signaling contribute to innate immune memory and infection control [39]. Surface-exposed proteins also play a central role in host–pathogen interaction, and their conservation across pathogenic species makes them key targets for vaccines aimed at heterologous protection [18,42].

Effective clearance of infection ultimately may depend on the activation of robust cellular immunity. High-resolution studies have demonstrated coordinated interactions between the γδ T-cell receptor and the WC1 co-receptor during *Leptospira* antigen recognition, leading to strong proliferation and IFN-γ production [22,58]. This axis drives a Th1/IgG2-skewed response, which may be essential for bacterial clearance from tissues and prevention of the chronic carrier state [22,56]. In contrast, bacterins formulated with aluminum salts predominantly induce a Th2/IgG1-biased humoral response [20,66,67].

To address these limitations, vaccine development has increasingly focused on conserved outer membrane and surface-exposed proteins identified through reverse and structural vaccinology approaches [23,33,68]. These methodologies enable the identification of antigens based on conservation, subcellular localization, and epitope content.

Among the most studied candidates are LipL32, LigA, LigB, and Loa22, which are expressed by pathogenic *Leptospira* species and exhibit high conservation across strains [33,69,70]. Experimental studies in ruminants have demonstrated that recombinant proteins such as LipL32, LigAni, and LigBrep are capable of inducing strong IgG responses, with repetitive Lig domains showing enhanced immunogenicity [71]. The development of chimeric constructs, such as LigAni–LigBrep (LC), further improves antigenicity and broadens immune recognition [52].

Nevertheless, humoral responses alone can be insufficient. Strategies such as heterologous prime–boost immunization, combining DNA and recombinant protein platforms, have shown promise in enhancing both antibody and T-cell-mediated immunity. These approaches are particularly relevant for overcoming the limited immunogenicity of isolated recombinant proteins and for promoting long-lasting immune memory [52,72,73,74].

Furthermore, adjuvant selection plays a decisive role in shaping these responses. While aluminum hydroxide remains widely used, its limited capacity to induce Th1 responses has prompted the exploration of alternative systems [48,66]. Oil-based emulsions such as Montanide, saponins, and biodegradable polymers like PLGA nanoparticles have demonstrated superior ability to enhance antigen presentation and promote Th1/IgG2 polarization [50,67,73]. TLR agonists, including monophosphoryl lipid A (MPLA), further potentiate these effects by mimicking pathogen-associated molecular patterns (PAMPs) and driving cellular immunity [48,67]. Advanced delivery platforms, including nanoparticles, liposomes, carbon nanotubes, and attenuated bacterial vectors, also contribute to improved antigen stability, targeted delivery, and controlled release [21,73].

Importantly, vaccine efficacy in leptospirosis cannot be attributed solely to adjuvant selection. Evidence from commercial bacterins indicates that factors such as antigen composition, strain selection, culture conditions, and preservation of native protein conformation critically influence immunogenicity and protective outcomes [38,74]. This is further supported by observations that, although some bacterins can reduce renal colonization, they fail to prevent genital colonization, particularly in BGL [13,19]. Additionally, multivalent formulations, while expanding serovar coverage, do not overcome the fundamental limitation of serovar-specific immunity and must be continuously updated to reflect regional epidemiology [21,75].

Emerging antigen classes, including virulence-modifying (VM) proteins (PF07598) and leucine-rich repeat (LRR), containing proteins, represent promising additions to the library of vaccine candidates. These targets are involved in pathogenesis and have demonstrated protective efficacy in experimental models, including reduction in tissue colonization [18,27,51]. VM proteins have also been detected in biological fluids of infected hosts, suggesting potential dual roles as diagnostic biomarkers and vaccine candidates [27]. However, a critical limitation remains the reliance on small animal models, such as hamsters and mice, whose disease progression differs significantly from that observed in ruminants, particularly regarding genital colonization [76].

Finally, the transition from classical bacterins to recombinant and subunit vaccines represents an important paradigm shift, from controlling clinical disease to targeting infection persistence and transmission. While conventional vaccines remain indispensable for reducing acute disease burden, they are insufficient for achieving herd-level control [77]. Recombinant strategies offer a path toward broader and more precise immunity, particularly when combining conserved antigens such as LipL32, LigA, and Loa22 with optimized adjuvants and delivery systems [33,78,79].

**Figure 1:**
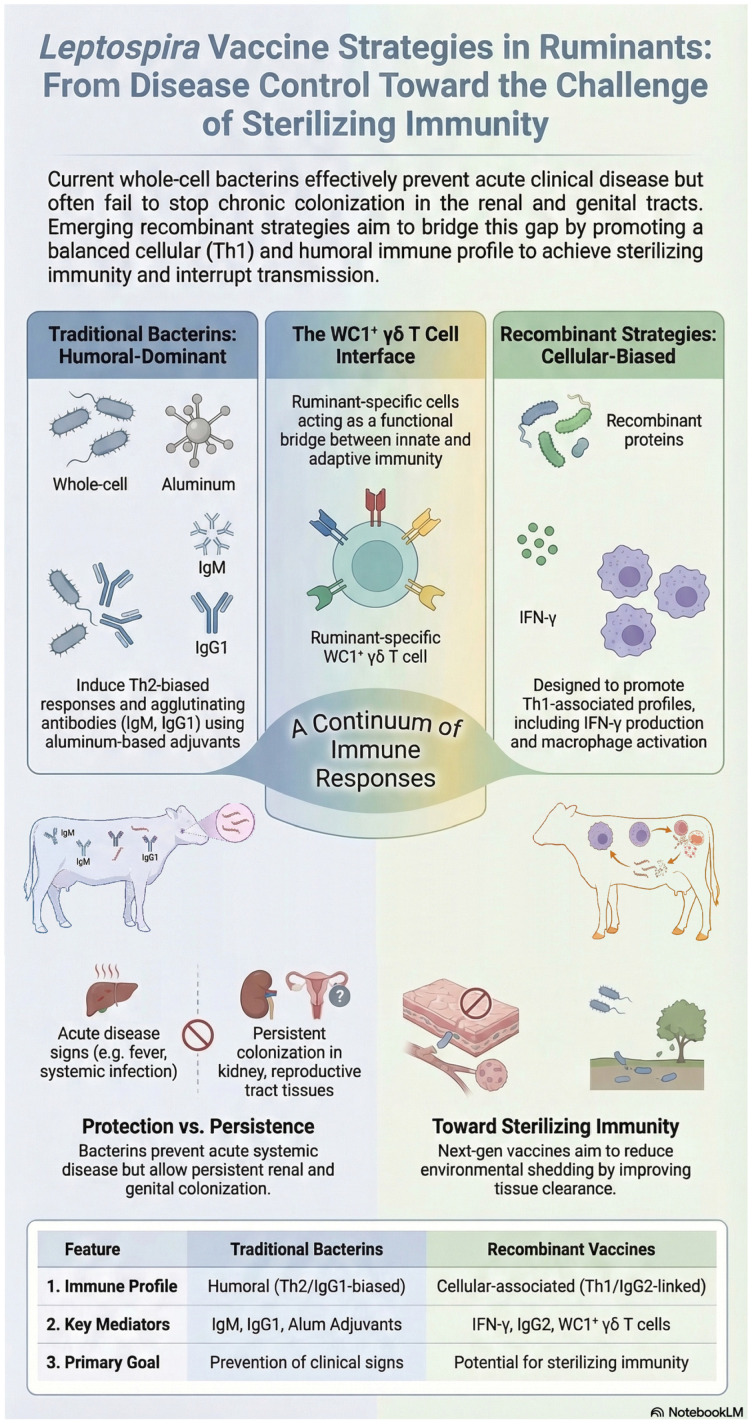
Immune mechanisms associated with protection and tissue persistence in bovine leptospirosis and their implications for vaccine design. This infographic was generated using Google NotebookLM (Google LLC—Mountain View, CA, USA) and subsequently reviewed and edited by the authors.

Ultimately, achieving sterilizing immunity in leptospirosis will require the integration of antigen discovery, adjuvant engineering, and delivery technologies capable of inducing coordinated humoral and cellular responses at relevant mucosal and tissue sites. The primary challenge lies not only in identifying protective antigens but in translating experimental success into field-applicable vaccines. This transition, from symptom control to interruption of transmission, represents the central goal for the next generation of leptospiral vaccines and is essential to mitigate both zoonotic risk and production losses [21,78]. These concepts are visually summarized in Figure 1 and further contextualized in Table 2 through a comparative analysis of classical bacterins and recombinant vaccines.

## 5. Mucosal Immunity in Ruminants and Strategies for Mucosal Vaccines Against BGL

Mucosal immunity also represents the first line of defense against the entry of *Leptospira*, which most frequently invades the host through mucosal surfaces. The bovine mucosal immune system is composed of mucosa-associated lymphoid tissue (MALT), which coordinates local and systemic responses [80].

Mucosal surfaces represent the primary portal of entry for *Leptospira,* and mucosal immunity represents the first line of defense against the disease. The bovine mucosal immune system, coordinated by MALT, provides a compartmentalized response that is often not adequately stimulated by systemic parenteral vaccination [80].

In the context of BGL and renal colonization, inducing a robust local response can be important, as systemic parenteral vaccines generally fail to generate protective levels of secretory IgA (sIgA) and resident memory T cells on mucosal surfaces [74,80]. Thus, the use of mucoadhesive delivery systems and adjuvants that mimic bacterial signals (such as MPLA and CpG) can overcome the mucosal tolerance barrier and induce a vigorous local response [48,67,81].

Studies suggest that the activation of γδ T cells, which are abundant in the mucous membranes of ruminants, through local stimuli, can accelerate bacterial elimination and prevent the establishment of the carrier state [53]. In ruminants, although resident mucosal populations are predominantly WC1−, it is established that WC1^+^ γδ T cells, capable of rapid IFN-γ production, can be recruited to these sites during the early stages of infection, potentially preceding even neutrophils [82,83,84].

While direct evidence for mucosal *Leptospira* vaccines in cattle is limited, successes in other veterinary contexts provide a potential roadmap [85,86]. For instance, intranasal and oral delivery of spore-based vaccines (e.g., *Bacillus subtilis* spores) against *Mannheimia haemolytica* in cattle has been shown to induce strong sIgA responses in bronchoalveolar lavage, saliva, and nasal secretions [87].

These examples suggest that the bovine MALT is highly receptive to organized antigen delivery, provided the physiological barriers are overcome. To achieve this in leptospirosis, innovative delivery systems are required to navigate the complex ruminant physiology [74]. The use of Lactic Acid Bacteria (LAB), such as *Lactobacillus plantarum* and *Lactococcus lactis*, as live vectors, offers a promising strategy. These “Generally Recognized as Safe” (GRAS) organisms act as natural adjuvants, protecting antigens from degradation and facilitating targeted delivery to the gut-associated lymphoid tissue (GALT). Preliminary evidence in animal models suggests that *L. plantarum* can modulate systemic inflammation and recruit myeloid cells early in the infection, potentially limiting the severity of leptospiral pathogenesis [88].

Beyond attenuated bacterial vectors, the use of viral vectors (e.g., adenoviruses), DNA vaccines, and mucoadhesive nanoparticles is being explored to bridge the gap between systemic and local immunity [18,78]. For example, DNA vaccines expressing chimeras like LC may be optimized for mucosal delivery to provide the necessary stimulus to protect against chronic colonization [52]. Nevertheless, these strategies must overcome significant hurdles, including the risk of immunological tolerance and the need for adjuvants that remain stable within the mucosal environment [74].

In conclusion, transitioning from parenteral to mucosal-targeted strategies, perhaps by combining conserved antigens like Loa22 with delivery systems that activate MALT, may increasingly be viewed as an avenue for achieving sterilizing immunity. However, such strategies remain experimental and require validation specifically within the context of leptospiral infection and ruminant reproductive physiology [18,51,80].

## 6. Conclusions

Leptospirosis vaccination in ruminants has reached a level of effectiveness in preventing clinical symptoms and inducing seroconversion, but the main gap remains the development of a vaccine that confers long-lasting sterilizing immunity, eliminating the renal and genital carrier state. While Th1-mediated cellular responses and WC1^+^ γδ T cells appear to be important correlates of protection, a more analytical and cautious approach is required when interpreting experimental data across different animal models. Recent evidence suggests that bovine γδ T cells can develop memory-like characteristics, including *Leptospira*, while for other stimuli, they have been shown to have trained immune responses. These could potentially amplify local immune responses after vaccination. This concept of trained immunity may explain inconsistencies between antibody titers and sterilizing protection and provides a new framework for vaccine design focused on preventing chronic carrier states. Future studies integrating cytokine profiling and epigenetic analyses will be essential to determine whether trained immunity contributes to protection against leptospiral colonization in ruminants, prioritizing the identification of specific protective antigens, the development of effective delivery systems, and the rigorous validation of these strategies in preventing the persistent carrier state in both renal and genital tissues. Thus, obtaining a vaccine with high sterilizing potential will allow vaccination to act not only as an animal health tool but also as a public health intervention, reducing economic losses in livestock farming and interrupting the zoonotic transmission chain.

## Figures and Tables

**Table 1:** Comparative overview of Th1 and Th2-directed cellular and humoral immune responses to bovine leptospirosis vaccination.

Feature/Goal	Humoral Axis (Th2/IgG1-Biased)	Cellular Axis (Th1/IgG2-Biased)
Primary Mediators	Agglutinating antibodies (IgM, IgG1), B-cell activation	IFN-γ, IgG2, WC1^+^ γδ T cells, activated macrophages
Target Pathogen Phase	Acute leptospiremia (bloodstream)	Chronic persistence (renal &/or genital tissues)
Clinical Outcome	Prevention of acute clinical signs and systemic dissemination	Potential for “sterilizing immunity” and reduction in environmental shedding
Serovar Context	Highly effective against incidental serovars (e.g., Pomona)	Critical for host-adapted serovars (e.g., Hardjo) and preventing the carrier state
Common Adjuvants	Aluminum hydroxide (Alum)	Saponins, VM proteins, biodegradable polymers, or DNA-prime/protein-boost
Trained Immunity Potential	Limited evidence for antibody-mediated “training” in this context	High potential involving γδ T cells, macrophages, NK cells, and NOD2 pathways
Overall Goal	Economic protection (reducing abortions/clinical illness)	Epidemiological control (interrupting the transmission cycle)
Feature/Goal	Humoral axis (Th2/IgG1-biased)	Cellular axis (Th1/IgG2-biased)
Primary Mediators	Agglutinating antibodies (IgM, IgG1), B-cell activation	IFN-γ, IgG2, WC1^+^ γδ T cells, activated macrophages, CD4^+^ T cells
Target Pathogen Phase	Acute leptospiremia (bloodstream)	Chronic persistence (renal &/or genital tissues)

**Table 2:** Comparison of classical bacterins and recombinant vaccines in bovine leptospirosis vaccination.

Feature	Classical Bacterins	Recombinant/Subunit Vaccines
Antigenic Basis	Whole-cell (inactivated)	Specific proteins (e.g., LigA, LipL32)
Breadth of Evidence in Cattle	Extensive (Field & Experimental)	Limited (Primarily Experimental)
Immune Profile	Primarily Humoral (IgG1)	Potential for Th1/IgG2/Cellular
Duration of Immunity	Short (typically <1 year)	Hypothesized to be longer
Renal Protection	Variable; can reduce shedding	Lacking cattle data

## Data Availability

No new data were created or analyzed in this study. Data sharing does not apply to this article.
